# Investigation of the prevalence and clinical implications of *ERBB2* exon 16 skipping mutations in Chinese pan-cancer patients

**DOI:** 10.3389/fonc.2022.1064598

**Published:** 2023-01-06

**Authors:** Yanhong Shang, Jianming Mo, Ran Huo, Xiaofang Li, Guotao Fang, Zichun Wei, Guomin Gu, Xiaodan Zhu, Chan Zhang, Chunling Liu, Dong Yan

**Affiliations:** ^1^ Department of Medical Oncology, Affiliated Hospital of Hebei University, Hebei Key Laboratory of Cancer Radiotherapy and Chemotherapy, Baoding, China; ^2^ Department of Pulmonary and Critical Care Medicine, Peking University Shenzhen Hospital, Shenzhen, China; ^3^ Thoracic Surgery, Peking University Shenzhen Hospital, Shenzhen, China; ^4^ Second Department of Pulmonary Medicine, Affiliated Cancer Hospital of Xinjiang Medical University, Urumqi, China; ^5^ Department of Oncology, Beijing Luhe Hospital, Capital Medical University, Beijing, China; ^6^ Department of Pulmonary Medicine, Affiliated Cancer Hospital of Xinjiang Medical, Urumqi, China

**Keywords:** deltaHER2, ERBB2, ERBB2delta16, exon 16 skipping, lung cancer

## Abstract

**Background:**

Although rare, *ERBB2* exon 16 skipping mutations (*ERBB2*ΔEx16) have been implicated in resistance to anti-HER2 and anti-EGFR targeted agents. Our study investigated the prevalence and clinical significance of *ERBB2*ΔEx16 in Chinese pan-cancer patients.

**Methods:**

We retrospectively screened 40996 patients, spanning 19 cancer types, who had available genomic profiles acquired with DNA-based next-generation sequencing (NGS). We characterized the clinical and molecular features of the *ERBB2*ΔEx16-positive patients. Furthermore, we also analyzed a pan-cancer dataset from the Cancer Genome Atlas (TCGA; n=8705).

**Results:**

A total of 22 patients were detected with *ERBB2*ΔEx16, resulting in an overall prevalence rate of 0.054% (22/40996). Of them, 16 patients had lung cancer (LC; 0.05%, 16/30890), five patients had gastric cancer (GC; 0.35%, 5/1448), and one patient had ovarian cancer (0.12%, 1/826). Among the 16 LC patients, *ERBB2*ΔEx16 was detected in four treatment-naïve *EGFR/ALK*-negative patients and 12 *EGFR*-positive patients after the onset of resistance to EGFR tyrosine kinase inhibitors (TKIs). The treatment-naïve patients harbored no LC-associated oncogenic drivers except *ERBB2* amplification, suggesting a potential oncogenic role for *ERBB2*ΔEx16. Consistently, *ERBB2*ΔEx16+ patients from TCGA data also carried no known drivers despite various concurrent alterations. In the 12 EGFR TKI-resistant LC patients, relative variant frequencies for *ERBB2*ΔEx16 were lower than in untreated patients, suggesting *ERBB2*ΔEx16 as secondary alterations following TKI treatment and thereby implicating *ERBB2*ΔEx16 in mediating therapeutic resistance.

**Conclusions:**

Our study identified an overall *ERBB2*ΔEx16 prevalence rate of 0.054% and provided insights into the clinical implications of *ERBB2*ΔEx16 in Chinese pan-cancer patients.

## Introduction

The Erb-B receptor tyrosine kinase 2 (HER2 or *ERBB2*) is a member of the Erb-B family and structurally related to the epidermal growth factor receptor (EGFR) (1). HER2 is a 185 kDa transmembrane receptor that lacks a ligand-binding structure and acts as a co-receptor by forming more potent heterodimers with HER1/EGFR and HER3/ErbB3 (1–5). Through its critical role in regulating cell growth and development, dysregulation of HER2 signaling, particularly HER2 overexpression, is one of the oncogenic drivers in various solid malignancies (1, 2, 4, 5). In addition to HER2 overexpression, genetic mutations affecting the extracellular, transmembrane, and kinase domains of *ERBB2* have been reported as alternative mechanisms of HER2 activation in various solid tumors and affect tumor biology and treatment response (5–8). Genetic alterations affecting the exon 16 of *ERBB2* (*ERBB2*ΔEx16) result in alternative splicing, lead to exon 16 skipping, and produce an altered HER2 protein that lacks 16 amino acids in the extracellular domain (amino acid positions 634-649) (5, 9, 10). *ERBB2*ΔEx16 is comprised of short in-frame deletions affecting exon 16 and missense mutations in splice donor or acceptor sites flanking exon 16. By altering gene splicing, the *ERBB2*ΔEx16 isoform could result in molecular conformational change by exposing cysteine residues crucial in intermolecular disulfide bond formation and lead to the constitutive activation and stable covalent binding of HER2 homodimers with more enhanced transformational activity (9–14).

*ERBB2*ΔEx16 was first reported in HER2-overexpressed breast cancer after prolonged targeted treatment with trastuzumab (5, 10–12). Preclinical studies in cell and mouse models have demonstrated the constitutive activation of *ERBB2*ΔEx16 and its critical role in tumorigenesis of breast and lung (9–15). Clinical studies have identified an *ERBB2*ΔEx16 prevalence of 0.01-0.19% in various solid tumor types (16, 17). Albeit rare, *ERBB2*ΔEx16 was implicated mostly in resistance to anti-HER2 agents in HER2-positive breast tumors and osimertinib in *EGFR*-mutant non-small-cell lung cancer (NSCLC) (5, 12, 16, 18). A deeper molecular understanding of the underlying pathogenesis of cancers could pave avenues for improving the treatment and survival outcomes of patients with *ERBB2*ΔEx16-positive (*ERBB2*ΔEx16+) tumors. In a large-scale analysis of Chinese cancer patients, Shi et al. identified 0.046% *ERBB2*ΔEx16+ cases (18/38680) spanning lung, colorectal, gastric, and ovarian cancers (19). Our study investigated the prevalence and clinical significance of *ERBB2*ΔEx16 in Chinese pan-cancer patients (n = 40996). To further characterize their mutational landscape and aberrant pathways, we also analyzed *ERBB2*ΔEx16-positive patients screened from pan-cancer datasets from the Cancer Genome Atlas (TCGA; n=8705) (20, 21).

## Patients and methods

### Patients

We retrospectively screened 40996 Chinese patients, spanning 19 cancer types, who voluntarily submitted either tissue or plasma samples for DNA-based next-generation sequencing (NGS) using either 168- or 520-gene panels between January 2018 to December 2020. We analyzed the clinical and molecular profile of the patients detected with *ERBB2*ΔEx16. For comparison, we obtained the molecular and survival data of a pan-cancer dataset from the TCGA (n=8705) (20, 21). This study was IRB approved and conducted in accordance with the ethical guidelines including Declaration of Helsinki and US Common Rule.

### Targeted next-generation sequencing

DNA was isolated from blood and tumor samples and subjected to NGS in Burning Rock Biotech, a clinical laboratory accredited by the College of American Pathologists and certified by the Clinical Laboratory Improvement Amendments, according to optimized protocols as described previously (22, 23). NGS library construction required a minimum of 30 ng of DNA. Target capture was performed using commercial panels consisting of either 168 or 520 cancer-related genes, which respectively span 0.269 Megabases (Mb) and 1.003 Mb of the human genome (Burning Rock Biotech, Guangzhou, China) (24, 25). Indexed samples were sequenced on NovaSeq 6000 (Illumina, Inc., CA, USA) with 150-bp read lengths and a target sequencing depth of 1,000× for tissue samples and 10,000× for plasma samples. Maximum allelic frequency (MaxAF) was defined as the maximum allelic frequency detected from a sample, and relative allelic frequency (RAF) was calculated as the ratio of the allelic frequency of a certain variant to MaxAF.

### Sequence data analysis

Sequence data were analyzed using the Burning Rock analysis system as previously described (22, 23, 26). Briefly, sequence data were mapped to the reference human genome (hg19). Variants with depth <100 or population frequency >0.1% in major databases were excluded from further analysis. Copy number was calculated based on the ratio between the depth of coverage in tumor samples and average coverage of an adequate number (n>50) of samples without CNV as references per capture interval. The cut-offs for copy number variations were 1.5 for copy number deletion and 2.5 for copy number amplifications.

### Statistical analysis

Statistical analyses were performed using the Wilcoxon signed-rank test or Fisher’s exact test as appropriate in R software (version 4.0.2). A two-sided P value <0.05 was considered statistically significant.

## Results

### Pan-cancer prevalence of ERBB2ΔEx16 alterations

**Figure 1:**
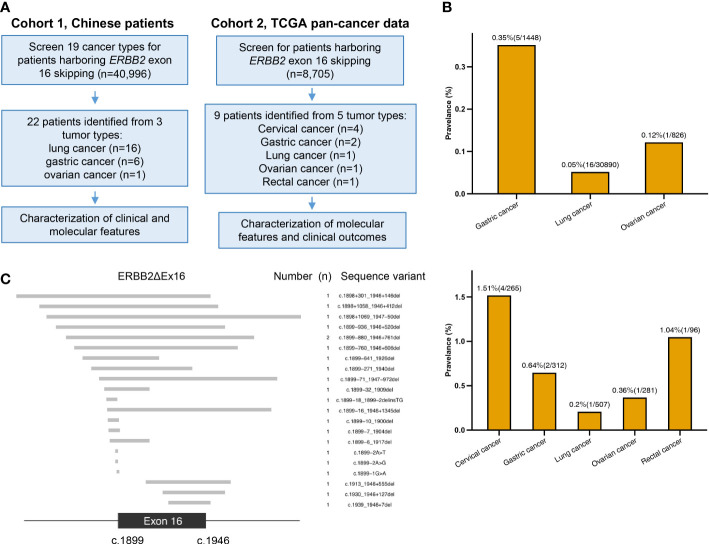
Study design and somatic *ERBB2* exon 16 skipping (*ERBB2*ΔEx16) analyzed in this study. **(A)** A diagram of study workflow. **(B)** Number of *ERBB2*ΔEx16+ patients from Cohorts 1 (upper panel) and 2 (lower panel) per tumor type, shown above the bars are the tumor type-specific prevalence (number of *ERBB2*ΔEx16+ patients/number of screened patients). **(C)** Diagram of the 22 unique sequence variants detected in the Chinese cancer patients cohort. The exon 16 (between nucleotides 1899 to 1946) and the flanking regions are shown, in which the gray bars correspond to each unique *ERBB2*ΔEx16 isoform as shown by the sequence variant on the right column.

**Figure 1A** illustrates the study design. We started with screening 40996 Chinese patients spanning 19 cancer types, most of which were lung cancer (LC; 71.4%), for patients who harbored *ERBB2*ΔEx16 (**Table S1**). A total of 22 patients were detected with *ERBB2*ΔEx16, resulting in an overall prevalence rate of 0.054% (22/40996). **Table 1** summarizes their clinical characteristics. The majority were female (54.5%) and had stage IV disease (60.9%). Most had LC (0.05%, 16/30,890), five patients had gastric cancer (GC; 0.35%, 5/1,448), and one patient had ovarian cancer (OC; 0.12%, 1/826). *ERBB2*ΔEx16 was not detected in other cancer types, including colorectal and breast.

**Table 1:** Clinical characteristics of the 22 patients detected with *ERBB2* exon 16 mutations in Cohort 1.

Characteristics	Overall (n = 22); n(%)	Lung (n = 16), n(%)	Non-lung (n = 6), n(%)
**Age**			
Median [interquartile range]	63.0 [51.0, 71.0]	65.5 [59.3, 71.0]	46.0 [41.0, 62.0]
**Stage**			
II	1 (4.5)	1 (6.2)	0 (0.0)
III	5 (22.7)	5 (31.2)	0 (0.0)
IV	13 (59.1)	10 (62.5)	3 (50.0)
NA	3 (13.6)	0 (0.0)	3 (50.0)
**Sex**			
Female	12 (54.5)	10 (62.5)	2 (33.3)
Male	9 (40.9)	6 (37.5)	3 (50.0)
NA	1 (4.5)	0 (0.0)	1 (16.7)
***ERBB2* exon16 mutation type**			
Exon 16 deletion	9 (40.9)	4 (25.0)	5 (83.3)
Splice site deletion	10 (45.5)	10 (62.5)	0 (0.0)
Splice site mutation	3 (13.6)	2 (12.5)	1 (16.7)
**Treatment status**			
Post-TKIs	12 (54.5)	12 (75.0)	0 (0.0)
Post-treatment	2 (9.1)	0 (0.0)	2 (33.3)
Primary	4 (18.2)	4 (25.0)	0 (0.0)
NA	4 (18.2)	0 (0.0)	4 (66.7)

NA, data not available.

We also screened 34 TCGA pan-cancer datasets (n = 8705) and identified nine *ERBB2*ΔEx16+ patients (**Figure 1B**). Of them, four patients had cervical squamous cell carcinoma and endocervical adenocarcinoma, two patients had stomach adenocarcinoma, and a patient each had lung adenocarcinoma, OC, and rectum adenocarcinoma. Clinical characteristics of the group and each patient are respectively detailed in **Tables S2 and S3**.

Of the 21 unique *ERBB2*ΔEx16 variants detected from Chinese patients, 9 involved complete deletion of exon 16, 3 were deletions or point mutations involving splice donors, and 9 deletions or point mutations affecting the splice acceptors (**Figure 1C**). *ERBB2* c.1899-880_1946+761del was detected from two patients. The other *ERBB2*ΔEx16 variants were unique and only detected in a patient each (**Table 2**). We also identified a novel variant *ERBB2* c.1899-2A>G, which was the only mutation detected from the paired tissue and plasma samples of an OC patient after treatment (P17; **Table 2**; **Figure 1C**).

**Table 2:** Detailed clinical and mutational profile of the 22 patients detected with *ERBB2* exon 16 alterations in Cohort 1.

Patient ID	Age	Sex	Clinical stage	Sample type	Cancer type	Treatment status	NGS capture panel	TMB	*ERBB2* exon 16 alterations	*ERBB2* exon16 mutation type	*ERBB2* exon16 mutant AF	*ERBB2* amp CN	Concurrent oncogenic mutations (AF or CN)
P01	70	Male	IV	Plasma	Lung	Post-TKIs	168-panel	NA	c.1898+1069_1947-50del ^#^	exon 16 deletion	0.74%	9.5	*EGFR* L858R(4.53%)
P02	57	Male	IV	Plasma	Lung	Post-TKIs	168-panel	NA	c.1913_1946+555del ^#^	splice site deletion	99.00%	7	*EGFR* L858R(5.42%)
P03	71	Female	IV	Plasma	Lung	Post-TKIs	520-panel	14.3	c.1899-32_1909del ^#^	splice site deletion	2.40%	32.32	*EGFR* L858R(43.08%); *EGFR* T790M(0.15%); *ERBB2* D769Y(2.02%); *ERBB2* L755S(1.63%)
P04	77	Female	III	Tissue	Lung	Primary	520-panel	3.99	c.1899-6_1917del ^#^	splice site deletion	2.94%	4.1	NA
P05	62	Female	IV	Plasma	Lung	Post-TKIs	168-panel	NA	c.1899-936_1946+520del ^#^	exon 16 deletion	13.20%	5.4	*EGFR* 19del(15.99%); *MET*_amp (CN 4.9); *MET* Y1230H(4.45%); *MET* D1228N(1.90%); *MET* L1195I(1.31%); *MET* L1195V(0.83%)
P06	74	Female	IV	Plasma	Lung	Post-TKIs	168-panel	NA	c.1899-271_1940del ^#^	splice site deletion	0.41%	4.2	*EGFR* L858R(12.01%); *EGFR* T725M(5.61%); *EGFR* R776C(12.88%)
P07	71	Male	IV	Tissue	Lung	Primary	520-panel	12	c.1899-2A>T	splice site mutation	49.11%	3.2	NA
				Plasma				1	c.1899-2A>T		0.91%	NA	NA
P08	64	Male	III	Tissue	Lung	Primary	520-panel	19.1	c.1899-7_1904del ^#^	splice site deletion	27.14%	3	NA
P09	63	Female	II	Plasma	Lung	Post-TKIs	168-panel	NA	c.1899-641_1926del ^#^	splice site deletion	5.31%	2.7	*EGFR* 19del(14.77%); *EGFR* T790M(2.24%); *EGFR* C797S(1.82%)
P10	68	Female	IV	Plasma	Lung	Post-TKIs	168-panel	NA	c.1939_1946+7del ^#^	splice site deletion	0.98%	2.7	*EGFR* L858R(34.39%); *EGFR* T790M(9.64%); *EGFR*_amp(CN 2.6):
				Tissue				NA	c.1939_1946+7del ^#^		11.86%	3.8	*EGFR* L858R(62.81%); *EGFR* T790M(16.47%); *EGFR*_amp(CN 3.3):
P11	51	Female	III	Tissue	Lung	Post-TKIs	168-panel	NA	c.1898+301_1946+146del ^#^	exon 16 deletion	99.00%	52.2	*EGFR* L858R(89.25%); *EGFR* L62R(89.56%)
P12	74	Male	IV	Tissue	Lung	Post-TKIs	520-panel	11	c.1899-16_1946+1345del ^#^	exon 16 deletion	6.05%	37.8	*EGFR* L858R(66.14%); *EGFR*_amp (CN 3.6); *ROS1*_fusion(37.65%)
P13	67	Female	III	Tissue	Lung	Primary	168-panel	NA	c.1899-18_1899-2delinsTG ^#^	splice site deletion	5.94%	NA	NA
P14	46	Male	IV	Plasma	Lung	Post-TKIs	168-panel	NA	c.1899-1G>A	splice site mutation	0.54%	2.7	*EGFR* 19del(1.17%)
P15	60	Female	III	Plasma	Lung	Post-TKIs	168-panel	NA	c.1899-10_1900del ^#^	splice site deletion	0.49%	3.19	*EGFR*_amp(CN 2.53); *EGFR* 19del(16.76%)
P16	49	Female	IV	Plasma	Lung	Post-TKIs	168-panel	NA	c.1930_1946+127del ^#^	splice site deletion	11.25%	3.1	*EGFR* L858R(67.39%); *EGFR* T790M(4.33%); *EGFR* E709K(66.23%); *EGFR*_amp(CN 3.5); *MET*_amp(CN 4.3); *RET*_fusion(1.67%); *ALK*_fusion(8.74%); *BRAF* V600E(0.68%)
P17	62	Female	IV	Plasma	Ovary	Post-treatment	520-panel	1	c.1899-2A>G ^#^	splice site mutation	2.06%	NA	NA
				Tissue				1	c.1899-2A>G ^#^		36.72%	NA	NA
P18	46	Female	NA	Plasma	Gastric	NA	168-panel	NA	c.1899-880_1946+761del ^#^	exon 16 deletion	0.98%	11.4	NA
P19	74	Male	NA	Tissue	Gastric	NA	520-panel	14	c.1899-760_1946+606del ^#^	exon 16 deletion	48.81%	12.1	NA
P20	34	Male	IV	Plasma	Gastric	Post-treatment	520-panel	23.9	c.1898+1058_1946+412del ^#^	exon 16 deletion	0.94%	25	NA
P21	41	Male	IV	Tissue	Gastric	NA	520-panel	4.99	c.1899-71_1947-972del ^#^	exon 16 deletion	2.21%	15.7	NA
P22	NA	NA	NA	Tissue	Gastric	NA	168-panel	NA	c.1899-880_1946+761del ^#^	exon 16 deletion	6.64%	18.7	NA

Pound signs (#) denote previously unreported sequence variants. Abbreviations: NA, data not available or not applicable; AF, allele frequency; amp, amplification; CN, copy number; 19del exon 19 deletion; TKI, tyrosine kinase inhibitor; TMB, tumor mutation burden (mutations/Mb of panel).

### Genomic profiles of ERBB2ΔEx16+ patients suggested potential roles in tumorigenesis and resistance to EGFR TKI in LC

**Figure 2:**
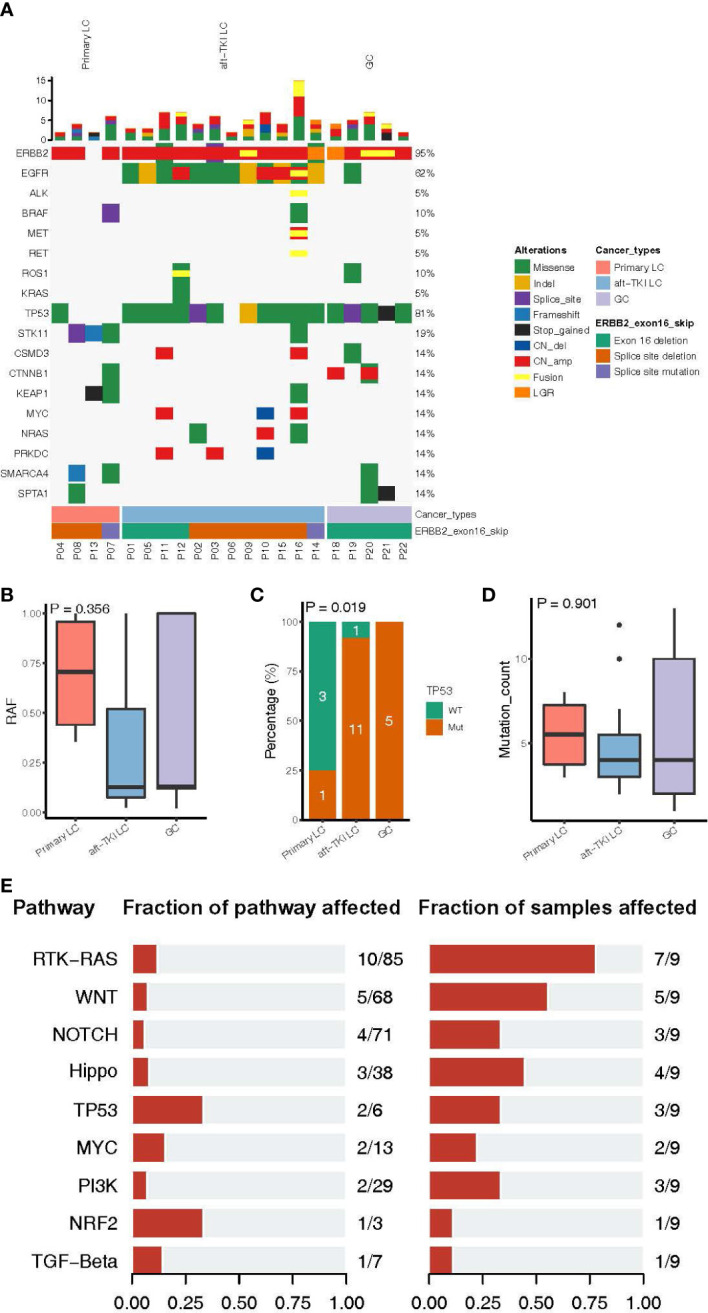
Molecular characteristics of patients harboring *ERBB2* exon 16 skipping (*ERBB2*ΔEx16). **(A)** An oncoprint of somatic mutation landscape of *ERBB2*ΔEx16+ patients from Cohort 1. Each row represents a gene indicated on the left, with the mutation rate indicated on the right. Each column represents a patient. Different colors denote the mutation types. Bar plots on top of the oncoprint summarize the number of mutations each patient carries. The cancer type, *ERBB2*ΔEx16 variant type, and patient ID of each patient were indicated by various colors at the bottom. **(B)** Relative allele frequency (RAF), **(C)** mutation frequencies of *TP53*, and **(D)** number of somatic mutations in lung cancer (LC) patients before systemic therapy, LC patients after progression on EGFR tyrosine kinase inhibitors (TKIs), and in gastric cancer (GC) patients. **(E)** Genomic alterations harbored by *ERBB2*ΔEx16+ patients from TCGA were categorized by pathway and analyzed for the proportion of mutated among all pathway-related genes and the corresponding mutation frequency in patients. Number pairs in the middle (e.g. “10/85” for the RTK-RAS pathway), indicate the number of genes encoding for members of the indicated pathway that were found altered in *ERBB2*ΔEx16+ patients and number of genes encoding for members of the pathway, respectively. Number pairs at the rightmost (e.g. “7/9” for the RTK-RAS pathway), indicate the number of *ERBB2*ΔEx16+ patients carrying ≥1 alteration in genes in the indicated pathway and the number of *ERBB2*ΔEx16+ patients, respectively.

Next, we characterized the clinical and molecular features of the 22 Chinese *ERBB2*ΔEx16+ patients. Among the 16 LC patients, 12 were *EGFR*-positive and had progressed on EGFR tyrosine kinase inhibitor (TKI) therapy (75.0%) and four were *EGFR/ALK*-negative and treatment-naïve (25.0%; **Table 2**). All *ERBB2*ΔEx16+ patients had concurrent *ERBB2* gene amplification except P08 (94.0%, 15/16; **Figure 2A**). Among the four previously untreated patients, three also harbored *ERBB2* amplification or *STK11* point mutations, two harbored *SMARCA4* or *KEAP1* point mutations, and one carried mutated *TP53, BRAF*, or *CTNNB1* (**Figure 2A**). No oncogenic driver gene mutations were detected from these four patients except *ERBB2* amplification, suggesting mutual exclusivity between *ERBB2*ΔEx16 and established drivers, and therefore a potential oncogenic role of *ERBB2*ΔEx16 in LC. This potential tumor-promoting activity was also supported by higher relative variant frequencies (RAFs) of *ERBB2*ΔEx16 detected in untreated LC patients than in their EGFR TKI-resistant counterparts or in GC patients (**Figure 2B**). The higher RAFs suggested an increased likelihood for *ERBB2*ΔEx16 to be clonal in the untreated lung tumors and therefore more likely to function as an oncogenic driver in LC. Consistent with our observation in Chinese patients, *ERBB2*ΔEx16+ patients from TCGA datasets also harbored no oncogenic drivers despite various concurrent alterations (**Figure S1**).

In addition to RAF, there were differences among the three patient subgroups in other molecular features. Treatment-naïve *ERBB2*ΔEx16+ LC patients were the least likely to carry concurrent *TP53* alterations (**Figure 2C**) and had the highest number of somatic mutations (**Figure 2D**). Analysis of genomic profiles from the TCGA data revealed scarce concurrent oncogenic driver alterations (**Figure S1**). Consistently, grouping co-occurring aberrations by pathway revealed likely aberrant signaling cascades in *ERBB2*ΔEx16+ patients, the most frequently of which receptor tyrosine kinase (RTK)-Ras (7/9 patients), WNT (5/9 patients), and Hippo (4/9 patients) signaling pathways (**Figure 2E**). Although their significance in tumor biology is well documented, the roles of these cascades in promoting transformation or conferring therapeutic resistance still await more clinical evidence. Taken together, the landscape of concomitant genomic alterations implicated *ERBB2*ΔEx16 in mediating oncogenesis and EGFR-TKI resistance and warrants further clinical evidence for validation.

Among the Chinese *ERBB2*ΔEx16+ patients, 12 had *EGFR*-positive LC and had been previously treated with EGFR TKI(s), accounting for 0.068% of the patients carrying *EGFR* sensitizing mutations (n = 17753). The lower RAFs suggested *ERBB2*ΔEx16 as a subclonal and/or secondary aberration in these EGFR TKI-resistant patients (**Figure 2B**). All 12 harbored concurrent *ERBB2* amplification and one had *MET* amplification and fusion (**Figure 2A**). The predominant majority (91.7%, 11/12) also harbored *TP53* mutations, which was in stark contrast with the treatment-naïve LC patients (**Figure 2C**). While *ERBB2* and *MET* amplification had been reported to confer resistance to EGFR TKI, their activities do not exclude the possibility of *ERBB2*ΔEx16 contributing to therapeutic resistance.

### Case vignettes

**Figure 3:**
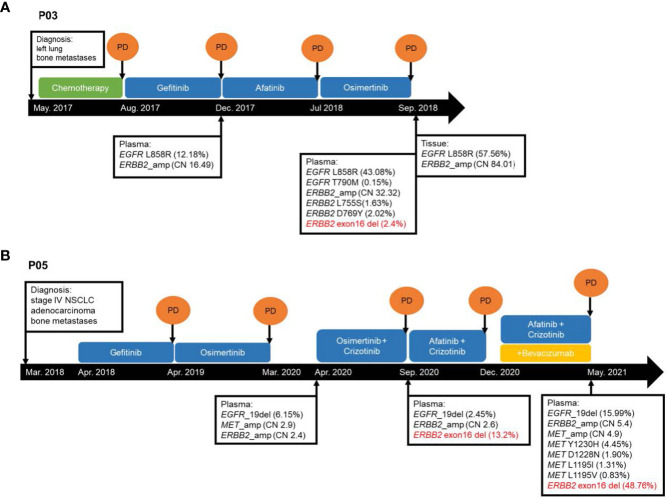
Schematic presentation of the courses of management for two *ERBB2*ΔEx16+ lung cancer patients **(A)** P03 and **(B)** P05 from Cohort 1. CN, copy number. NSCLC, non-small cell lung cancer. PD, progressive disease.

P03 was a female patient with *EGFR* L858R-mutant advanced LUAD with bone metastasis. *ERBB2*ΔEx16 was detected after disease progression with osimertinib using her plasma samples but not in the paired tissue rebiopsy (**Table 2**; **Figure 3A**). Prior to fourth-line osimertinib, the patient had received first-line chemotherapy, followed by sequential EGFR TKIs, including second-line gefitinib and third-line afatinib monotherapies. In addition to *EGFR* L858R, her plasma sample collected after osimertinib progression was also detected with other concurrent *ERBB2* alterations, including *ERBB2*ΔEx16 (c.1899-32_1909del), gene amplification (copy number: 32.32), and L755S and D769Y (27), suggesting one or more of these alterations as resistance mechanisms.

P05 was a female patient with *EGFR* exon 19 deletion-mutant stage IV LUAD with bone metastasis, and *ERBB2*ΔEx16 was identified from her plasma sample after progression on osimertinib plus crizotinib (**Table 2**; **Figure 3B**). She had received first-line gefitinib and second-line osimertinib and was subsequently detected with *MET* amplification after the second disease progression. After onset of resistance to third-line osimertinib plus crizotinib, her plasma sample was detected with *EGFR* exon 19 deletion, *ERBB2*ΔEx16 (c.1899-936_1946+520del), and *ERBB2* amplification. She was then treated with afatinib and crizotinib but with no clinical benefit, and bevacizumab was subsequently added to the regimen. Upon progression, molecular testing revealed increased levels of *ERBB2* amplification (copy number: 2.6 to 5.4) and *ERBB2*ΔEx16 (VAF from 13.2% to 48.8%) as well as various *MET* alterations, including amplification, Y1230H, D1288N L1195I, and L1195V, the latter four of which are known secondary mutations associated with MET TKI resistance (28, 29). Similar to patient P03, one or more of these genetic abnormalities may have conferred resistance to the targeted agents. Together, these cases illustrate the potential role of *ERBB2*ΔEx16 in mediating EGFR TKI resistance and disease progression in LC.

## Discussion

Through screening a large real-world population, our findings demonstrated an overall *ERBB2*ΔEx16 prevalence of 0.054% (22/40996) among Chinese cancer patients. Our findings were consistent with another real-world study that reported a prevalence of 0.046% (18/38,680) (19). Importantly, almost all the in-frame deletions identified in our study were unique and were absent from the Catalogue of Somatic Mutations in Cancer (COSMIC) database. Of the 21 unique *ERBB2*ΔEx16 from our cohort, *ERBB2* c.1899-1G>A was detected from a patient each with LC and GC. *ERBB2* c.1899-1G>A was previously reported in breast, lung, and gastric cancer (16, 30). Moreover, *ERBB2* c.1899-2A>T with concurrent *ERBB2* gene amplification were the mutations detected from baseline tumor and plasma samples of a stage IV LC patient (P07). This variant was also identified in an LC patient in the TCGA datasets and a previous study (16). Contrastingly, *ERBB2* c.1899-2A>G, a different transition located at the same splice site, is absent from the COSMIC database. We identified this variant from an OC patient (P17) as the only potentially oncogenic mutation in paired tumor (520-gene panel) and plasma (168-gene panel) samples, which suggested a tumorigenic role for this *ERBB2*ΔEx16 variant.

The oncogenic activity of *ERBB2*ΔEx16 was first suggested in breast tumors (10). Compared with *ERBB2* wild-type tumors, *ERBB2*ΔEx16+ mammary tumors exhibit a higher degree of intratumoral heterogeneity as shown by distinct signaling and gene expression profiles associated with the activation of tumor initiation and progression (14, 31). Subsequent preclinical studies have established the critical role of *ERBB2*ΔEx16 in the increased aggressiveness of HER2-positive breast tumors (9–14, 31). Turpin et al. also demonstrated intrinsic resistance of *ERBB2*ΔEx16+ breast cancer cell lines to the antibody-drug conjugate ado-trastuzumab emtansine (T-DM1) due to the lack of internalization of the T-DM1-*ERBB2*ΔEx16 complex, which is crucial for T-DM1 efficacy (14). Although *ERBB2*ΔEx16 has been implicated in trastuzumab resistance in breast cancer, the role of *ERBB2*ΔEx16 in mediating inhibitor resistance remains controversial (32). Numerous studies have also reported trastuzumab sensitivity of cell samples from patients with HER2-positive breast and gastrointestinal cancer that express *ERBB2*ΔEx16 (9, 17, 31, 33). In our cohort, *ERBB2*ΔEx16 was not detected among the 778 women with breast cancer included in the screening population. We speculate that *ERBB2*ΔEx16 is rare in Chinese women with breast cancer and requires a larger cohort for its detection.

In LC, Smith et al. have also demonstrated the transforming activity of *ERBB2*ΔEx16 *in vitro* and *in vivo* (15). *ERBB2*ΔEx16 has been implicated in osimertinib resistance *in vitro* (18). Interestingly, we also observed that all but one *ERBB2*ΔEx16+ patients also had concurrent *ERBB2* gene amplification (95.5%, 21/22). All 12 *EGFR*-positive LC patients detected with *ERBB2*ΔEx16 at progression on EGFR-targeted therapy also harbored concurrent *ERBB2* amplification. As a well-documented resistance mechanism of EGFR inhibitors, *ERBB2* amplification was found in 10-15% patients with acquired resistance to first- or second-generation EGFR-TKIs (34) and 2-5% of patients with acquired resistance to first- (35) or second-line osimertinib (36). In the study by Shi et al, 10 of the 12 *ERBB2*ΔEx16+ LC patients also harbored *ERBB2* amplification, and although it was unknown whether 2 wild-type patients had been treated (19). Despite its presence, *ERBB2* amplification (present in 2.9% patients with sensitizing *EGFR* mutations, 508/17753) did not exclude potential roles of the ΔEx16 mutant in mediating drug resistance. Moreover, the allele frequencies (AFs) of the ΔEx16 allele were both 99.0% in patients P02 and P11 (**Table 2**), both of whom were LC patients with acquired resistance to EGFR-TKI. These findings were consistent with a tumor-promoting role for the *ERBB2*ΔEx16 mutant. For the remaining patients, it is still possible that the ΔEx16 allele was the predominantly amplified allele, although the low AFs and amount of the wild-type allele from the non-malignant cells present in the sequenced samples did not allow for a definitive conclusion. Further research, including those using preclinical models that express abnormally high copies of *ERBB2*ΔEx16 and those analyzing the AFs for the ΔEx16 allele from circulating tumor cells, may unravel the significance of concurrent *ERBB2* amplification and exon 16 skipping in conferring TKI resistance.

Additionally, *ERBB2*ΔEx16 was also detected without *ERBB2* amplification in a treatment-naïve LC patient and an OC patient, neither of whom carrying LC-associated oncogenic driver alterations. *ERBB2*ΔEx16 has been previously found in a patient with low grade serous ovarian tumor (37) and one OC patient (19). In the latter patient, no mutations in known cancer driver genes were detected. Together, these and our findings supported tumor-promoting roles of *ERBB2*ΔEx16 in LC and OC.

Preclinical and clinical investigations have shown the differential activity of some irreversible pan-HER TKIs such as afatinib, dacomitinib, and neratinib in targeting various *ERBB2* mutant alleles across cancer types (6–8, 38, 39). A subset of patients with breast, lung, or cervical cancer demonstrated promising clinical outcomes with pan-HER TKIs; however, the same inhibitors were inefficacious in other tumor types, including colorectal and bladder cancer (8). Various treatment strategies for HER2-mutant cancers are actively investigated in different phases of clinical trials (8). Since *ERBB2*ΔEx16 only affects the extracellular domain, resulting in a protein product with an intact tyrosine kinase domain, pan-HER TKIs could be efficacious. Tilio et al. reported that the *ERBB2*ΔEx16+ breast cancer cell lines were resistant to lapatinib but were sensitive to dacomitinib (40). Hsu et al. also reported a patient with *EGFR* L858R/T790M-positive advanced LC who acquired *ERBB2*ΔEx16 during osimertinib therapy (18). The authors also reported that afatinib was able to reverse *ERBB2*ΔEx16-mediated osimertinib resistance in *EGFR* L858R/T790M double mutant LC cell line (18). Our patient (P05) received afatinib combined with crizotinib after the emergence of *ERBB2*ΔEx16. However, no clinical benefit was observed, which may have been due to the intratumoral genetic heterogeneity. The clinical efficacy of pan-HER inhibitors and other novel treatment strategies in inhibiting *ERBB2*ΔEx16-mediated signaling in a certain subset of patients with *ERBB2*ΔEx16+ tumors deserves further investigation. It also remains to be explored whether other concurrent genomic alterations, such as *TP53* mutations, could affect treatment response. *TP53* is the most commonly co-mutated gene in *ERBB2*ΔEx16+ tumors in our cohort. The presence of co-occurring genetic alterations that affect treatment responses, such as *ERBB2*ΔEx16, *ERBB2* gene amplification, or *TP53* mutations, highlights the importance of elucidating the molecular profile of baseline and multiple rebiopsy samples to monitor treatment-related mutational changes and optimize treatment decisions.

Due to the retrospective nature of our study, clinical, treatment, and survival outcomes for some patients are not available, which severely limits our analysis. Our study did not use RNA-based analysis to investigate whether the *ERBB2*ΔEx16 variants detected in our cohort could result in alternative splicing.

## Conclusion

Our study identified an overall *ERBB2*ΔEx16 prevalence rate of 0.054% and provided an insight into the rarity of *ERBB2*ΔEx16 in Chinese pan-cancer patients. Among patients with LC, *ERBB2*ΔEx16 was detected before receiving treatment and after developing resistance from EGFR TKIs, suggesting its potential role in inhibitor resistance. Our study also raises the need to develop novel drugs and implement novel therapeutic strategies to improve the survival outcomes of patients with *ERBB2*ΔEx16.

## Data availability statement

The datasets presented in this study can be found in online repositories. The names of the repository/repositories and accession number(s) can be found below: NODE (National Omics Data Encyclopedia) OEP003749.

## Ethics statement

The studies involving human participants were reviewed and approved by IRB approved and conducted in accordance with the ethical guidelines including Declaration of Helsinki and US Common Rule. The patients/participants provided their written informed consent to participate in this study.

## Author contributions

YS and JM conceived of the study and drafted the manuscript. YS, JM, CL, and YD collected data, analyzed and interpreted the data, and drafted the manuscript. CL and YD provided supervision and administrative support. All authors contributed to the article and approved the submitted version.

## Supplementary material

Supplementary Figure 1 An oncoprint summarizing the somatic mutation landscape of the nine *ERBB2*ΔEx16+ patients arranged according to signaling pathway. The groups of genes within the gray box are genes related to the signaling pathway indicated at the bottom of the gray border (i.e. the first 5 rows of the oncoprint are genes related to Wnt signaling pathway as noted in the sixth row at the bottom of the gray border). Each row represents a gene or signaling pathway indicated on the left, with the mutation rate indicated on the right. Each column represents a patient. The bar plot on top of the oncoprint summarizes the number of mutations each patient carries. Different colors denote the alteration types. Dark gray denotes mutated signaling pathways. The tumor type was indicated by various colors at the bottom of the oncoprint.
