# Supplementary material for: Investigation of the prevalence and clinical implications of ERBB2 exon 16 skipping mutations in Chinese pan-cancer patients
Source: Front Oncol. 2023 Jan 6;12:1064598. doi: 10.3389/fonc.2022.1064598 (PMC9859631; doi:10.3389/fonc.2022.1064598)
Supplement: Supplementary file 1 [file DataSheet_1.docx]

**Table S1**. List of the 19 cancer types screened for *ERBB2* exon 16 mutations for Cohort 1

| Cancer types | N=33,786  n (%) |
| --- | --- |
| Lung | 24,115 (71.4) |
| Colorectal | 3,034 (9.0) |
| Gastric | 1,313 (3.9) |
| Ovarian | 1,178 (3.5) |
| Breast | 778 (2.3) |
| Lymphoma | 451 (1.3) |
| Pancreatic | 434 (1.3) |
| Urinary bladder | 382 (1.1) |
| Prostate | 343 (1.0) |
| Endometrial | 274 (0.8) |
| Bile duct | 259 (0.8) |
| Liver | 227 (0.7) |
| Sarcoma (non-gastrointestinal stromal tumor) | 222 (0.7) |
| Kidney | 219 (0.6) |
| Cervical | 196 (0.6) |
| Head and neck | 117 (0.3) |
| Melanoma | 104 (0.3) |
| Gastrointestinal stromal tumor | 84 (0.2) |
| Thyroid | 56 (0.2) |
