# Supplementary material for: Investigation of the prevalence and clinical implications of ERBB2 exon 16 skipping mutations in Chinese pan-cancer patients
Source: Front Oncol. 2023 Jan 6;12:1064598. doi: 10.3389/fonc.2022.1064598 (PMC9859631; doi:10.3389/fonc.2022.1064598)
Supplement: Supplementary file 2 [file DataSheet_2.docx]

**Table S2**. Clinical characteristics of the TCGA cohort

| Characteristics | ERBB2ΔEx16-positive (n = 9) | Cervical squamous cell carcinoma (CESC) (n = 307) | Lung adenocarcinoma (LUAD) (n = 522) | Ovarian cancer (OV) (n = 585) | Rectum adenocarcinoma (READ) (n = 170) | Stomach adenocarcinoma (STAD) (n = 443) |
| --- | --- | --- | --- | --- | --- | --- |
| Age; Median[IQR] | 50.0 [46.0, 57.0] | 46.0 [38.0, 56.5] | 66.0 [59.0, 73.0] | 59.0 [51.0, 68.0] | 66.0 [57.0, 72.0] | 67.0 [58.0, 73.0] |
| NA | 0 (0.0) | 0 (0.0) | 19 (3.6) | 0 (0.0) | 0 (0.0) | 5 (1.1) |
| Sex |  |  |  |  |  |  |
| Female | 5 (55.6) | 307 (100.0) | 280 (53.6) | 585 (100.0) | 78 (45.9) | 158 (35.7) |
| Male | 4 (44.4) | 0 (0.0) | 242 (46.4) | 0 (0.0) | 92 (54.1) | 285 (64.3) |
| Clinical stage |  |  |  |  |  |  |
| I | 3 (33.3) | 163 (53.1) | 0 (0.0) | 17 (2.9) | 0 (0.0) | 0 (0.0) |
| II | 0 (0.0) | 70 (22.8) | 0 (0.0) | 30 (5.1) | 0 (0.0) | 0 (0.0) |
| III | 2 (22.2) | 46 (15.0) | 0 (0.0) | 445 (76.1) | 0 (0.0) | 0 (0.0) |
| IV | 0 (0.0) | 21 (6.8) | 0 (0.0) | 89 (15.2) | 0 (0.0) | 0 (0.0) |
| NA | 4 (44.4) | 7 (2.3) | 522 (100.0) | 4 (0.7) | 170 (100.0) | 443 (100.0) |
| Overall survival (OS)status |  |  |  |  |  |  |
| Alive | 5 (55.6) | 236 (76.9) | 334 (64.0) | 236 (40.3) | 144 (84.7) | 271 (61.2) |
| Dead | 4 (44.4) | 71 (23.1) | 188 (36.0) | 349 (59.7) | 26 (15.3) | 172 (38.8) |
| OS time (days) |  |  |  |  |  |  |
| Median[IQR] | 1011.0 [426.0, 1108.0] | 636.0 [352.5, 1244.0] | 656.0 [416.0, 1130.0] | 1005.5 [469.75, 1674.0] | 609.5 [369.25, 1096.0] | 426.0 [225.0, 752.0] |
| NA | 0 (0.0) | 0 (0.0) | 9 (1.7) | 3 (0.5) | 0 (0.0) | 6 (1.4) |
| 5-year survival (95%CI) | 30% (6.3%-100%) | 65.9% (58.8%-73.9%) | 40.7% (34.4%-48.2%) | 34% (29.7%-39%) | 49.1% (32.4%-74.5%) | 37.6% (29.7%-47.6%) |

Abbreviations: CI, confidence interval; IQR, interquartile range; NA, data not available; OS, overall survival
