# Supplementary material for: Investigation of the prevalence and clinical implications of ERBB2 exon 16 skipping mutations in Chinese pan-cancer patients
Source: Front Oncol. 2023 Jan 6;12:1064598. doi: 10.3389/fonc.2022.1064598 (PMC9859631; doi:10.3389/fonc.2022.1064598)
Supplement: Supplementary file 3 [file DataSheet_3.docx]

**Table S3**. Detailed information of the nine patients from the TCGA cohort identified with *ERBB2* exon 16 mutations

| bcr_patient_barcode | Cancer type | Age | Sex | Race | Clinical stage | Overall survival (OS)status | Overall survival time (days) |
| --- | --- | --- | --- | --- | --- | --- | --- |
| TCGA-C5-A1M9 | CESC | 46 | Female | White | IB1 | Dead | 1065 |
| TCGA-C5-A2M2 | CESC | 56 | Female | White | IB2 | Dead | 1011 |
| TCGA-C5-A7X8 | CESC | 35 | Female | White | IB1 | Alive | 83+ |
| TCGA-MA-AA43 | CESC | 48 | Female | White | IIIB | Alive | 346+ |
| TCGA-05-4384 | LUAD | 66 | Male | NA | NA | Alive | 426+ |
| TCGA-25-1328 | OV | 38 | Female | White | IIIC | Dead | 2009 |
| TCGA-AH-6643 | READ | 50 | Male | White | NA | Dead | 1314 |
| TCGA-D7-8573 | STAD | 57 | Male | White | NA | Alive | 593+ |
| TCGA-D7-A4YX | STAD | 63 | Male | White | NA | Alive | 1108+ |

Note: Overall survival time with plus sign (+) denotes alive status as of the data cutoff.

Abbreviations: CESC, cervical squamous cell carcinoma and endocervical adenocarcinoma; LUAD, lung adenocarcinoma; NA, data not available; OS, overall survival OV, ovarian cancer; READ, rectum adenocarcinoma; STAD, stomach adenocarcinoma
