# Supplementary figures and images for: Investigation of the prevalence and clinical implications of ERBB2 exon 16 skipping mutations in Chinese pan-cancer patients
Source: Front Oncol. 2023 Jan 6;12:1064598. doi: 10.3389/fonc.2022.1064598 (PMC9859631; doi:10.3389/fonc.2022.1064598)

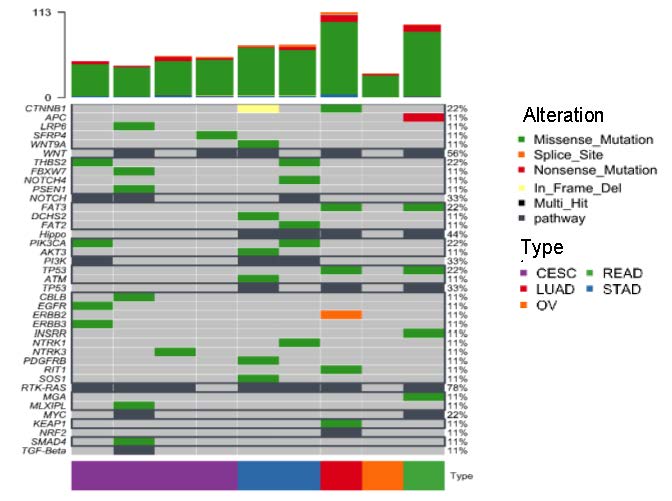

Supplement: Supplementary Figure 1 — An oncoprint summarizing the somatic mutation landscape of the nine ERBB2ΔEx16+ patients arranged according to signaling pathway. The groups of genes within the gray box are genes related to the signaling pathway indicated at the bottom of the gray border (i.e. the first 5 rows of the oncoprint are genes related to Wnt signaling pathway as noted in the sixth row at the bottom of the gray border). Each row represents a gene or signaling pathway indicated on the left, with the mutation rate indicated on the right. Each column represents a patient. The bar plot on top of the oncoprint summarizes the number of mutations each patient carries. Different colors denote the alteration types. Dark gray denotes mutated signaling pathways. The tumor type was indicated by various colors at the bottom of the oncoprint. [file Image_1.jpeg]
